# A molecular genetic study of autism and related phenotypes in extended pedigrees

**DOI:** 10.1186/1866-1955-5-30

**Published:** 2013-10-05

**Authors:** Joseph Piven, Veronica J Vieland, Morgan Parlier, Ann Thompson, Irene O’Conner, Mark Woodbury-Smith, Yungui Huang, Kimberly A Walters, Bridget Fernandez, Peter Szatmari

**Affiliations:** 1 Carolina Institute for Developmental Disabilities, University of North Carolina at Chapel Hill, School of Medicine, CB# 3367, Chapel Hill, NC 27599, USA; 2 Battelle Center for Mathematical Medicine, The Research Institute at Nationwide Children’s Hospital, 575 Children’s Crossroad, Columbus, OH 43215, USA; 3 Department of Pediatrics and Department of Statistics, The Ohio State University, 575 Children’s Crossroad, Columbus, OH 43215, USA; 4 McMaster Department of Psychiatry and Behavioural Neurosciences, 1200 Main Street west, L9H 3Z5, Hamilton, ON, Canada; 5 Provincial Medical Genetics Program, Health Sciences Center, 300 Prince Philip Drive, A1B 3V6, St. John’s, Newfoundland, Canada; 6 Centre for Addiction and Mental Health, University of Toronto, 80 Workman Way, Toronto, ON, Canada

**Keywords:** Autism, Genetics, Linkage, Pedigree, Phenotype, Posterior probability of linkage

## Abstract

**Background:**

Efforts to uncover the risk genotypes associated with the familial nature of autism spectrum disorder (ASD) have had limited success. The study of extended pedigrees, incorporating additional ASD-related phenotypes into linkage analysis, offers an alternative approach to the search for inherited ASD susceptibility variants that complements traditional methods used to study the genetics of ASD.

**Methods:**

We examined evidence for linkage in 19 extended pedigrees ascertained through ASD cases spread across at least two (and in most cases three) nuclear families. Both compound phenotypes (i.e., ASD and, in non-ASD individuals, the broad autism phenotype) and more narrowly defined components of these phenotypes, e.g., social and repetitive behavior, pragmatic language, and anxiety, were examined. The overarching goal was to maximize the aggregate information available on the maximum number of individuals and to disaggregate syndromic phenotypes in order to examine the genetic underpinnings of more narrowly defined aspects of ASD behavior.

**Results:**

Results reveal substantial between-family locus heterogeneity and support the importance of previously reported ASD loci in inherited, familial, forms of ASD. Additional loci, not seen in the ASD analyses, show evidence for linkage to the broad autism phenotype (BAP). BAP peaks are well supported by multiple subphenotypes (including anxiety, pragmatic language, and social behavior) showing linkage to regions overlapping with the compound BAP phenotype. Whereas 'repetitive behavior’, showing the strongest evidence for linkage (Posterior Probability of Linkage = 62% at 6p25.2-24.3, and 69% at 19p13.3), appears to be linked to novel regions not detected with other compound or narrow phenotypes examined in this study.

**Conclusions:**

These results provide support for the presence of key features underlying the complexity of the genetic architecture of ASD: substantial between-family locus heterogeneity, that the BAP appears to correspond to sets of subclinical features segregating with ASD within pedigrees, and that different features of the ASD phenotype segregate independently of one another. These findings support the additional study of larger, even more individually informative pedigrees, together with measurement of multiple, behavioral- and biomarker-based phenotypes, in both affected and non-affected individuals, to elucidate the complex genetics of familial ASD.

## Background

Autism spectrum disorder (ASD), a neurodevelopmental condition associated with lifelong disability, has now become an urgent public health challenge. Research into the genetics of ASD is motivated by the very real possibility that genetic testing can play a role in improving the diagnostic process and in providing targets for the development of new drugs [1]. However, much remains to be learned about the genetics of ASD before these goals are realized.

Early family studies based on retrospective data found a 2–9% recurrence of ASD among siblings of affected probands [2]. Recent work using prospective data and more up to date criteria [3] observed closer to 1 in 4 siblings affected – roughly 25 times higher than in the general population. Not only is the disorder familial, but variation in phenotypes such as IQ and language appear to be correlated among affected siblings as well [4-6]. Twin studies demonstrate that concordance for a phenotype that includes both autism and milder cognitive and social-communication deficits and rigidity (termed the broader autism phenotype or BAP), was >80% among monozygotic twins, compared with ~10% in dizygotic twin pairs [7], although the dizygotic rate is substantially higher in the most recent twin study [8]. This BAP also appears more common in relatives (particularly parents) of ASD probands than controls (20% vs. <10%) and more commonly in male than female relatives of ASD probands [9-11]. A recent prospective study of high-risk infant siblings indicates that this same extended phenotype (BAP traits, cognitive delays, and anxious behaviors) can be seen in upwards of 20% of infant siblings (who do not go on to develop ASD) compared to 3% in low-risk controls [12]. These combined findings indicate that ASD is a familial disorder (with roughly 40% of siblings showing either ASD or an extended BAP) with a strong genetic component.

However, studies attempting to uncover the risk genotype associated with the familial nature of the disorder have been largely unsuccessful. A review of linkage studies using mostly affected sibling pairs has indicated many 'significant’ linkage peaks with some replication among linkage signals [13]. Fine mapping under the peaks has not been successful in uncovering ASD genes, likely due to, among other reasons, a degree of allelic heterogeneity that, in retrospect, was greater than anticipated and perhaps by an over-reliance on the compound phenotype of ASD as opposed to a more modular approach. Four large independent genome-wide association studies have been reported, but so far the data indicate that few, if any, common variants have a substantial impact on risk [14-17]. A recent cross-disorder genome-wide association study reports more promising results, but specificity of those common variants to ASD is unclear [18].

In startling contrast, there is substantial evidence that a multitude of rare *de novo* copy number variants (CNVs) contribute, at least in part, to the etiology of sporadic (i.e., non-familial) ASD [19,20]. To quantify the role of CNVs, a series of large studies (for a review see [21]), have used microarrays to interrogate ASD cohorts. Although estimates vary among studies, between 2% to 10% of probands appear to have *de novo* structural variants [21,22]. Every risk locus or gene so far occurs with less than 1% frequency in ASD cases.

The field has been more likely to assign a causal role to *de novo* CNVs than to inherited ones in spite of the fact that ASD is a familial disorder and that rare inherited CNVs have also been consistently reported in ASD. Several pedigrees showing inherited CNVs or point mutations in a number of key CNS genes or regions (SHANK1, CDH8, NRXN3, PTCHD1, 16p11.2) have also been published [23-27]. In many of these, the transmitting parent had the BAP or a related phenotype; for example, in a pedigree transmitting a SHANK1 deletion, we determined that the transmitting parents (both mothers) had suffered from anxiety disorders [26] (such disorders were not present in non-transmitting relatives who we were able to assess). In other pedigrees, medical/congenital or cognitive abnormalities in relatives also appear to be segregating with a rare variant. However, these represent single case reports and it is therefore hard to argue that these phenotypes are variable expressions of the ASD genotype at the population level as opposed to the individual case level.

The study of extended pedigrees (defined here as including relatives outside of the nuclear family identified by an ASD proband) offers an opportunity to study the inherited nature of ASD more thoroughly. Such pedigrees may have enough information to be able to follow the segregation of a genetic variant through a single family to see if it is associated with phenotype status. Several groups have employed the extended pedigree strategy, ranging from a search for shared CNVs across cousin pairs to studying a single, extensive Finnish pedigree from 20 nuclear families [28-32]. One study incorporated measurement of the social responsiveness scale subphenotype into the analysis of extended pedigrees and concluded that this was a robust and useful approach for exploring genetic linkage in studies of ASD [30]. If an important goal is to understand the familial nature of ASD, a greater focus on inherited variants is warranted employing a broad range of phenotypes including those segregating in parents and siblings without ASD. However, there are no systematic linkage studies on whether non-ASD individuals with a potentially pathogenic variant have any phenotype of interest such as the BAP and related phenotypes.

Incorporating additional phenotypes into linkage analysis may be a robust way to uncover inherited ASD susceptibility variants. Consider a genetic mutation that increases the risk of ASD and which can also produce a sub-clinical phenotype in mutation carriers. Misclassifying carriers as 'unaffected’ artificially reduces penetrance estimates for linkage analysis, while misclassifying non-carriers as 'affected’ introduces apparent phenocopies; either of these errors will tend to depress linkage signals. Thus, in a set of pedigrees segregating this mutation, the more accurately the sub-clinical phenotype is measured, the stronger the linkage evidence should be. In other words, a clinical definition that correctly captures the segregation pattern of the mutation will, all other things being equal, result in stronger linkage evidence. Similarly, a quantitative trait (QT) that correctly segregates with the mutation will produce stronger linkage evidence, in general, than one that does not. Here, we utilize clinical data on several ASD-related phenotypes in conjunction with linkage analysis of extended pedigrees to explore genotype-phenotype relationships in ASD.

## Methods

### Participants

We recruited extended pedigrees with at least three ASD cases spread across at least two nuclear families (in all but one case, the three cases were spread across three nuclear families). All families were either known to the authors through previous studies or identified through advertising. In all, 19 families were available for this study, 6 recruited in Canada (CA) and 13 in the US. The CA pedigrees had an average of 24 genotyped individuals and 25 phenotyped individuals, while the US pedigrees had an average of 16 genotyped individuals and 18 phenotyped individuals. To minimize etiologic heterogeneity, families were excluded from the study if there was evidence of the following co-occurring medical conditions thought to be etiologically-related to autism in one of the index probands with autism: tuberous sclerosis, neurofibromatosis, phenylketonuria, Fragile X screening, or significant CNS injury. We did not exclude individuals with a chromosome abnormality in order to determine whether that abnormality might also be inherited and play a role in susceptibility. All individuals were of northern European heritage. All data collection took place under Institutional Review Board approval and the research was conducted in accordance with the World Medical Association Declaration of Helsinki [33]. Written informed consent was obtained from all subjects after the study had been fully explained.

### Clinical methods

Our overarching clinical strategy included clinical assessments performed to i) index eligible extended pedigrees, by identifying at least three related individuals with a Diagnostic and Statistical Manual (4^th^ ed) (DSM-IV) pervasive developmental disorder; and to ii) characterize all pedigree members on phenotypes of interest. For the latter goal of characterizing pedigree members, the strategy employed was to assess for both compound phenotypes (i.e., ASD or, in non-ASD individuals, the BAP) and more narrowly defined components of these phenotypes including social and repetitive behavior, pragmatic language, and anxiety. The overarching goal in taking this multi-tiered approach was to maximize the aggregate information available on the maximum number of affected individuals (i.e., global ratings of ASD or the BAP) as well as to disaggregate these global phenotypes to look at the genetic underpinnings of individual behavioral aspects of the broad construct of autistic behavior.

Overlapping sets of instruments were used to diagnose ASD and the BAP in the CA and US pedigrees, respectively. After initial screening based on the Telephone Screening Interview, the Autism Family History Interview [34], and review of medical records, diagnosis was based on expert clinical judgment incorporating information from the Autism Diagnostic Interview Revised [35] and Autism Diagnostic Observation Schedule – Revised [36], administered by trained and reliable clinicians. All participants classified as ASD met DSM IV criteria [37] for either Autistic disorder, Asperger syndrome, or pervasive developmental disorder not otherwise specified according to the criteria in Risi et al. [38]. The US extended pedigrees included 3 to 6 individuals with ASD (average = 4); while the CA extended pedigrees included 3 to 8 (average = 5).

Non-ASD family members were assessed for BAP, which broadly covers the domains of aloof personality, rigid personality, and pragmatic language deficits. The Modified Personality Assessment Schedule Revised (MPAS-R) [39,40] and Modified Pragmatic Rating Scale (MPRS) [39,40], which are the preferred methods for BAP assessment in individuals aged 16 or over, were administered only to the US families. The MPAS-R [41] is a semi-structured interview for rating personality characteristics adapted from the Personality Assessment Schedule [42], further revised to assess six personality characteristics: aloof, anxious, hypersensitive, overly-conscientious, rigid, and untactful [40]. In the MPAS-R, information is collected via separate self and informant interviews and the characteristics are rated as either present, absent or unknown [39] by two independent clinicians, with a third rater serving as a tiebreaker when needed. For this study, only two MPAS-R characteristics (aloof and rigid) were utilized in determining the BAP, although all characteristics were queried and rated to maintain the integrity of the instrument. Aloof and rigid have consistently been the most valid and reliable at distinguishing relatives of individuals with autism from relatives of typically developing individuals in our previous studies (e.g., Piven et al. [40]). The MPRS is an abbreviated version of the pragmatic rating scale (PRS) [43], developed to identify seven pragmatic language skills and four prosodic and grammatical speech errors with more efficiency and less redundancy than the PRS. These eleven items were extracted from the PRS using logistic regression to determine those that most reliably predict pragmatic speech deficits; they best differentiated parents of children with autism and parents of typically developing children in the Iowa Family Study [40], are highly correlated to the PRS, and were validated during the Collaborative Linkage Study of Autism [44]. Trained interviewers rate items 0, 1, or 2 based on a guided conversation incorporated in the MPAS-R that ensures that all rated behaviors have the opportunity to be observed. The PRS has good inter-rater reliability (κ = 0.77) [40,43].

When MPAS-R and MPRS consensus ratings were not available, as for all CA pedigrees and some US individuals, the BAP Questionnaire (BAP-Q) [45] was used for diagnosis of the BAP and components (aloof and rigid personality, and pragmatic language deficits). The BAP-Q was also used as a screening tool and mailed to participants to determine whether or not to follow-up with more comprehensive BAP assessment or to gather 'diagnostic’ information on participants who were otherwise unavailable for in-person visits. The BAP-Q is a self- and informant-report questionnaire consisting of 36 items spread across three 12-item subscales derived from direct assessment interviews (social aloofness and rigid personality from the MPAS-R and pragmatic language abnormalities from the MPRS). Items are rated along a six-point Likert scale (ranging from 'very rarely’ to 'very often’), which forces ratings to fall above or below a value of neutral on each question. Original internal consistency analysis of the subscales [45] supported this three-component model, which is consistent with the traditional conceptualizations of domains characterizing autism: social, communication and restricted and repetitive behaviors. The measure was completed by the participant about him/herself (the self-version) and by someone close to the participant about him/her (the informant version) to obtain an average score (between the self and informant scores); whenever available, the average scores were utilized. A BAP diagnosis was assigned if an individual met gender-specific criteria in any domain [46] (see Table 1 for cut-offs). These diagnostic cut-offs are higher than those originally published in 2007 and have higher specificity than the 2007 cut-offs now suggested for use in screening. The US pedigrees had a range of 2 to 10 BAP positive cases (average = 5); the CA pedigrees had a range of 1 to 6 BAP positive cases (average = 4). Note that when analyzing ASD alone, BAP positive individuals were coded as unaffected.

**Table 1:** Cut-off scores for BAP-Q self and informant ratings, and the average of the two, for males and females

	**Male**	**Female**
Aloof	Self: 4.13	Self: 3.45
	Informant: 4.19	Informant: 3.64
	Average: 4.03	Average: 3.39
Pragmatic language	Self: 3.23	Self: 2.94
	Informant: 3.29	Informant: 3.19
	Average: 3.09	Average: 2.90
Rigidity	Self: 3.91	Self: 3.70
	Informant: 4.20	Informant: 4.30
	Average: 3.90	Average: 3.85
Total score	Self: 3.55	Self: 3.17
	Informant: 3.63	Informant: 3.46
	Average: 3.47	Average: 3.19

Original 'best estimate’ cut-off scores reported by Hurley et al. [45] were as follows: Male: Aloof 3.25, Pragmatic Language: 2.95, Rigid: 3.65; Total Score: 3.35; Female: Aloof 3.00, Pragmatic Language: 2.70, Rigid: 3.25; Total Score: 3.25*.*

The US (but not CA) families were additionally assessed on seven secondary phenotypes. The secondary US phenotypes address various aspects of autism and include anxiety (ANX), repetitive/ritualistic behavior (RRB), social functioning (SOC), and language abnormalities, i.e., social communication (PRS), core language ability (CLF), non-word repetition (NWR), and rapid naming (RAP). The CL, NWR, and RAP phenotypes were considered exploratory as there is less support for these constructs showing familiality in the literature, and were added to enrich the overall assessment for communication phenotype information, one of three major criteria for autism in the DSM IV.

ANX, RRB, and SOC are quantitative variables. ANX is based on the anxiety (N1) facet t-score of the NEO Personality Inventory Revised [47], given to non-ASD family members aged 17 and over (reported to aggregate in parents of autistic probands [40]). Since ANX was not measured in ASD cases, a quantitative trait threshold model was used, designating ASD cases as 'over threshold’ and non-cases as under (when no ANX score was available). Observed ANX has a bell-shaped distribution with an overall mean score of 51 (SD = 9), and the within-pedigree means ranged from 47 to 63. RRB is the overall score from the Repetitive Behavior Scale Revised [48], and has an overall mean of 13 (SD = 16) and is higher in ASD cases (mean = 26, SD = 16) than non-cases (mean = 4, SD = 7). Mean pedigree RRB varies greatly from 6 to 31. SOC is the total t-score from the Social Responsiveness Scale [49]. The SOC distribution is skewed right with an overall mean of 55 (SD = 18) and is also greater in ASD cases (mean = 84, SD = 14) than non-cases (mean = 48, SD = 11). Mean pedigree SOC is fairly stable with a range of 48 to 67.

The remaining variables are dichotomous. PRS is based on ASD status and the social score of the MPRS, used above to diagnose BAP; 61% of those assessed with MPRS were positive on PRS and 24% of non-ASD cases were positive on PRS. All ASD positive individuals were considered as affected for PRS. The pedigrees have between 6 and 10 PRS cases. CLF affection indicates a standard core language score from the Clinical Evaluation of Language Fundamentals 4^th^ Edition below 84 (1 SD below the mean) [50]. Half of ASD cases were positive on CLF while only 2% of non-cases were positive. Families had at most three CLF cases. NWR and RAP are phenotypes derived from performance on the Comprehensive Test of Phonological Processing [51]: NWR cases have non-word repetition subtest standard scores ≤7 (1 SD below the mean), and RAP cases have rapid naming composite scores <84 (1 SD below the mean) [52]. For NWR, 41% of ASD cases were positive compared to 23% of non-cases. Similarly for RAP, 46% of ASD cases were positive versus 11% of non-cases. The extended pedigrees have at most 9 or 7 cases of NWR or RAP, respectively, but there are pedigrees with none for both phenotypes.

### Genotyping and data cleaning

Three hundred and twenty-two individuals (range 7–32, mean 17/pedigree) were genotyped using a dense microsatellite (MS) marker set (avg. intermarker distance 4 cM) obtained from DeCode; 280 individuals (range 6–29, mean 15/pedigree) were also genotyped using the Illumina OMNI 2.5 M chip. In preparation for linkage analysis, both MS and SNP data were used to verify family structure; founders were assessed for relatedness (no relatedness found), and extended relationships were confirmed. All genotypes were cleaned for marker missingness (dropping markers above thresholds of 5% for SNPs, 25% for MSs), sample missingness (>5% SNP; >25% MS), and excess Mendel errors both by marker and individual; SNPs with a Hardy-Weinberg *p* value <1x10^-10^ and MSs below 1x10^-4^ were dropped. After thinning of the SNP map to remove marker-marker linkage disequilibrium (R^2^ >0.20), we based linkage analyses on a combined map comprising 10,364 SNPs + 1,078 MSs. The genetic map was based on the Build 36 hg18 (Build 37 hg19 used for X only) Rutgers Combined Linkage-Physical Map (http://compgen.rutgers.edu/RutgersMap) [53] (custom release November 2011). The full set of (pre-thinned) SNPs was also used to call CNVs. No CNVs were observed to segregate within pedigrees and they are therefore not further discussed in this paper.

### Statistical methods

Linkage analysis was conducted using the software package Kelvin, which implements the PPL class of models for measuring the strength of genetic evidence [54,55]. (“PPL” originally stood for “posterior probablity of linkage.”) In order to take advantage of the very dense marker coverage and given the size of the pedigrees, MCMC was used to calculate marker likelihoods as described in [56], while Kelvin’s non-stochastic algorithm was used to calculate trait likelihoods conditional on marker data [57]. We note that these calculations are highly computationally intensive, requiring approximately 36,000 CPU hours (1-month real time) with calculations distributed over a 90-node Linux cluster with 16–128 Gb memory/node.

Three different trait models were employed, depending on the type of trait being analyzed: dichotomous trait (DT, used for ASD, BAP, CLF, NWR, PRS and RAP), quantitative trait (QT, used for RRB, SOC), and QT threshold (QTT, used for ANX). The DT model is parameterized in terms of α (the admixture parameter of Smith [58], representing the proportion of 'linked’ pedigrees), *p* (the disease allele frequency), and the penetrance vector *f*_*i*_, representing the probability that an individual with genotype *i* develops disease, for *i* – 1..3. The QT model replaces the penetrances with a vector of three genotypic means and three genotypic variances. Note that normality is assumed at the genotypic level, but not at the population level, and there is no inflation of scores under violations of normality [59]. The QTT model extends the QT model by allowing for 'affected’ individuals, who are missing QT values but assumed to have exceeded some threshold on the QT scale, with the threshold itself being an additional parameter of the model. All trait parameters are integrated out of the final statistic, using essentially uniform prior distributions (ordering constraints are imposed on the penetrances or means), and unbounded parameters are integrated over a finite range [54,59], implicitly allowing for dominant, recessive, and additive models. This provides a robust approximation for mapping complex traits in terms of the marginal model at each locus, and because the parameters are integrated out, no specific assumptions regarding their values are required.

The PPL has two basic approaches to the accumulation of evidence, which we employ here to consider evidence across pedigrees. Under 'pooled’ (PPL_POOL_), the trait parameters are integrated across all pedigrees as a set at each locus. This is appropriate under the expectation that at each locus, the trait model is essentially the same across pedigrees. Under 'sequential’ (PPL_SEQ_), trait parameters are integrated over separately for each pedigree at each locus, and the marginal evidence for or against linkage itself is accumulated across pedigrees using Bayesian sequential updating. Sequential updating is appropriate under the expectation that each pedigree may implicate different loci and/or the same loci but under different trait models (as could arise, e.g., in the presence of important background genetic and/or environmental modification). When there is relative genetic homogeneity, pooling will yield larger signals at linked loci; when there is extensive heterogeneity, however, sequential will yield larger signals at linked loci and also smaller signals at unlinked ones [60].

Since the PPL framework differs from standard frequentist statistical approaches, a few comments on its interpretation will be helpful. First, the PPL is on the probability scale, and its interpretation is therefore straightforward, e.g., PPL = 40%, means that there is an estimated 40% probability of a trait gene at the given location based on the data. The only caveat to this interpretation is that this estimated probability is influenced by the low prior probability of linkage (π) to any given locus. Based on empirical data [61] we set π = 2% (this assumes just one disease gene in the genome and is thus conservative, possibly highly conservative, under locus heterogeneity). Thus PPL >2% indicates (some degree) of evidence in favor of a trait gene at that locus, while PPL <2% represents evidence against the location. As with any Bayesian method, the influence of this small prior probability on the final PPL can be appreciable until the data set becomes large. Hence, even a small PPL – say, 20% – indicates that the data are supporting linkage enough to make the posterior probability 10 times larger than the prior. Hence, in finite samples, we do not interpret 1-PPL as the probability of no gene; rather, we interpret the PPL relative to the prior probability π = 2%.

Additional distinctive features of the statistical framework are related to the fact that the PPL is a measure of statistical evidence, not a decision-making procedure. There are, therefore, no 'significance levels’ associated with it (i.e., no specific cut-offs beyond which we declare significance) and it is not interpreted in terms of associated error probabilities [62,63]. By the same token, no multiple testing corrections are applied to the PPL, just as one would not 'correct’ a measure of the temperature made in one location for temperature readings taken at different locations [64].

## Results

The results are presented in four sections: i) we consider the comparison between pooled and sequentially updated results for both ASD and BAP, in order to gauge the extent of between-family heterogeneity, and then present primary ii) ASD and iii) BAP results, followed by iv) results for the secondary phenotypes.

### i) Pooling vs. sequential updating

**Figure 1:**
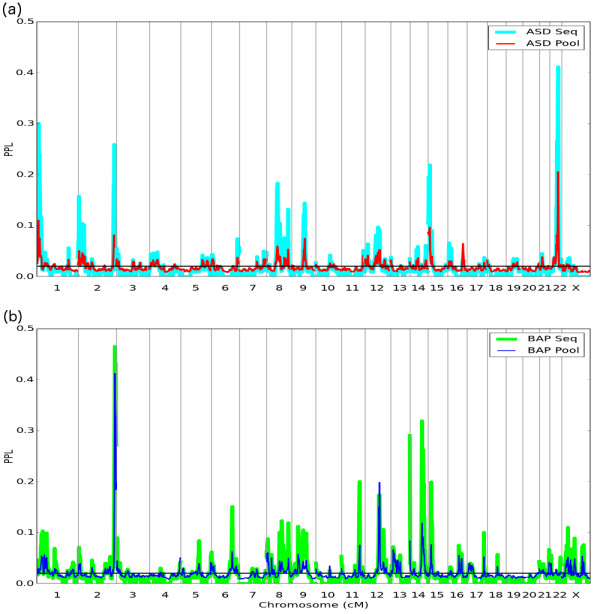
**Genome scans for (a) ASD and (b) BAP comparing 'pooled’ with 'sequentially updated’ results.** Note that for visual clarity, the y-axis goes from 0.0–0.5, rather than 0.0–1.0.

Figure 1a shows pooled and sequential results across the genome for ASD, while Figure 1b shows results for BAP. Within each sub-figure, results are correlated, as expected. However, for ASD, there was just 1 PPL_POOL_ ≥0.20, but 4 under sequential (max PPL_SEQ_ = 0.41, at the same position as PPL_POOL_ = 0.20); while for BAP the corresponding numbers were 1 (PPL_POOL_ = 0.41) and 5 (with max PPL = 0.46 at that same position). Across the board, where PPL >10%, PPL_SEQ_ > PPL_POOL_ (with one exception on chromosome 12 for BAP, where PPL_POOL_ = 0.20 and PPL_SEQ_ = 0.17). At the same time, the proportion of the genome showing evidence against linkage (PPL <2%) was 74% and 79% for ASD pooled and sequential, respectively, and 66% and 70% for BAP pooled and sequential, respectively. Thus, for both phenotypes, a greater proportion of the genome showed evidence against linkage under sequential analysis, and at these locations, the evidence against linkage tended to be stronger. This pattern of results is consistent with appreciable genetic heterogeneity across pedigrees. In what follows, we therefore rely on sequential as the primary data analytic approach to the accumulation of evidence going forward, dropping the 'SEQ’ subscript.

### ii) ASD results (sequential)

Four loci show PPL >20% (1p36.32, PPL = 30%; 2q37.2, PPL = 26%; 15q12, PPL = 22%; 22q13.31, PPL = 41%), with additional loci on chromosomes 2, 8, 9, and possibly 12 standing out above the background as well (Figure 1a). Not surprisingly, no single pedigree generates large PPLs on its own. We note however, that on 8q12.1 a modest sequential peak appears to be driven by a single pedigree (PPL = 19% in Ped 4); because the sequential peak is somewhat lower than this, we can conclude that the remaining pedigrees in aggregate show some evidence against linkage to this locus. On 15q11.2, a single pedigree (Ped 16) shows PPL = 15% on its own; however, the sequential peak is >15%, suggesting that while this one pedigree accounts for the preponderance of the evidence, one or more additional pedigrees must also be supporting linkage at this locus. By contrast, PPLs of comparable size on 16q23.1-q23.2 (PPL = 19% in Ped 16) and Xp22.11-p21.3 (PPL = 18% in Ped 5) are almost completely erased in the sequential results. This is consistent with the possibility of major loci within each individual pedigree not found in any of the remaining pedigrees.

### iii) BAP results (sequential)

**Figure 2:**
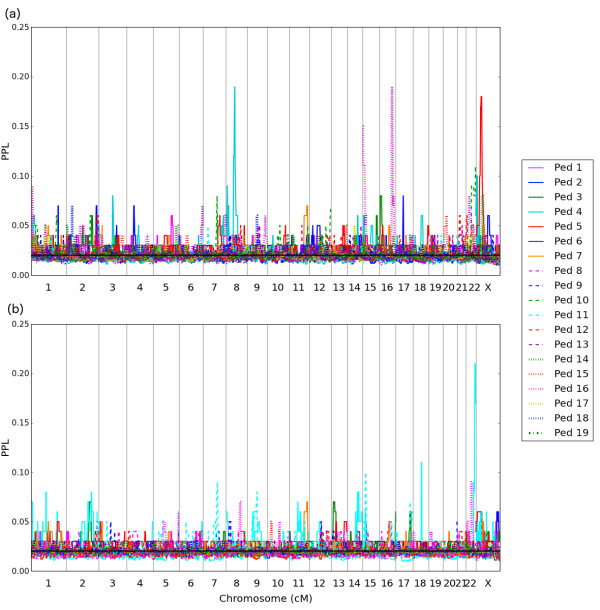
**Genome scans for (a) ASD and (b) BAP, by individual pedigree.** Note that the y-axis goes from 0.0–0.25.

Five additional loci, not seen in the ASD analyses, show BAP PPL >20% (Figure 1b): 2q37.3 (PPL = 47%), 11q23.3 (PPL = 20%), 14q11.2 (PPL = 29%), 14q31.3 (PPL = 32%) and 15q13.3 (PPL = 20%). Considering individual pedigrees (Figure 2b), just one salient peak emerges, and this is on 22q13.32 (PPL = 21% in Ped 4). Interestingly, while that locus was salient in the omnibus (all pedigrees) ASD scan, under ASD this particular pedigree gives evidence against linkage (PPL <2%) at this locus.

**Figure 3:**
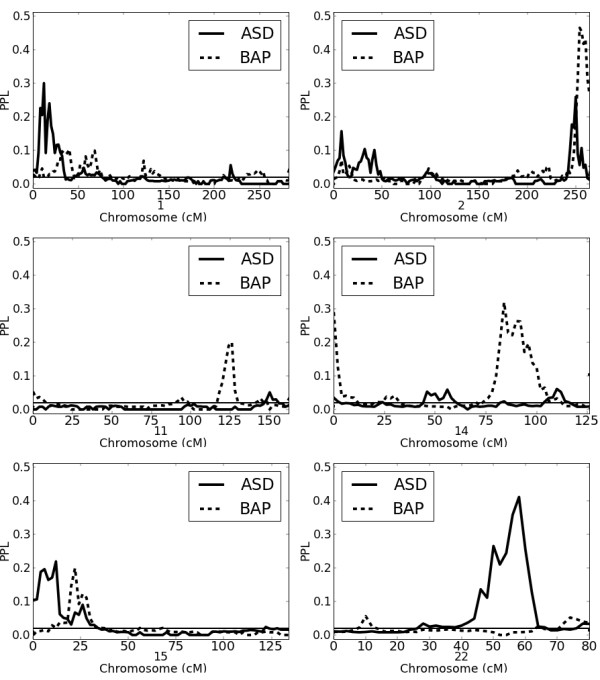
**ASD and BAP results for selected chromosomes.** Note that the y-axis goes from 0.0–0.5.

Table 2 shows salient results for both ASD and BAP; Figure 3 shows ASD and BAP plots for selected chromosomes. Not surprisingly, at most loci, results are correlated; at only 2 loci (11q23.2, 22q13.31) does one phenotype show evidence for and the other against linkage. Notably, of the remaining 5 loci, in only one case (1p36.32) is the ASD score substantially higher than the BAP score, while at 5 of 7 loci the BAP score is substantially higher than the ASD score.

**Table 2:** Primary ASD and BAP findings

**Locus**	**cM**^**a**^	**Phenotype**		**Individual pedigrees driving signals**
		ASD	BAP	
1p36.32	12	0.30	0.02	ASD PPL = 0.09, Ped 16
2q37.3	254	0.26^b^	0.47	
11q23.3	126	0.00	0.20	
14q11.2	0	0.04	0.29	
14q31.3	84	0.02	0.32	BAP PPL = 0.08, Ped 4
15q12	12	0.22	0.199^c^	ASD PPL = 0.15, Ped 16; BAP PPL = 0.10, Ped 11
22q13.31	58	0.41	0.01	ASD PPL = 0.09, Ped 10

^a^The cM position and locus correspond to the maximum PPL value in any peak >20%. Additional detail can be seen in Figure 3. ^b^The ASD peak is at 250 cM in 2q37.2. ^c^The BAP peak is at 22 cM in 15q13.3.

### iv) Results for additional phenotypes

**Figure 4:**
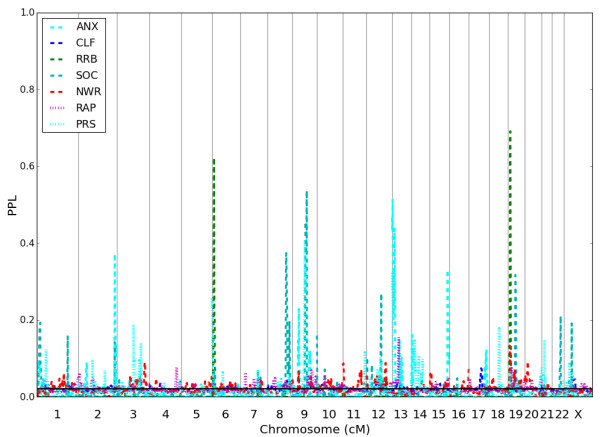
Genome scan for seven additional phenotypes, US pedigrees only.

Figure 4 shows genome-wide results for each of the seven secondary phenotypes (US pedigrees only). There are multiple loci with signals greater than any seen in the BAP analyses (with the exception of 2q37.2-q37.3, which is higher for BAP than any of the sub-phenotypes). None of the exploratory phenotypes NWR, RAP, or CLF shows any signals >15% genome-wide; these phenotypes are not further considered here. It is important to keep in mind that due to the limited size of the pedigrees, results across phenotypes are expected to show some degree of correlation, whether the phenotypes are measuring the same underlying traits or not. Another way to express this is to note that considering multiple phenotypes in a single set of pedigrees is a form of permuting the phenotypes; combined with selection of loci based on maximum scores, this will tend to lead to 'inflation’ of linkage results [65]. Nevertheless, bearing this caveat in mind, some interesting patterns emerge.

First, there are 4 loci at which BAP has the highest PPL (Table 3). Of these, perhaps surprisingly, 3 loci are either not supported or only marginally supported by any of the other phenotypes. Only 1 BAP peak (2q37.2-q37.3) is well supported by multiple subphenotypes, with only RRB giving evidence against linkage across this locus.

**Table 3:** Results for BAP and secondary phenotypes for any locus with PPL >20% for at least one phenotype (US pedigrees only)

**Locus**	**cM**^**a**^	**Phenotype**				
		**BAP**^**b**^	**ANX**	**PRS**	**RRB**	**SOC**
2q37.2-q37.3	248–264	0.59	0.37	0.08	0.00	0.14
6p25.3-p25.2	0–8	0.04	0.00	0.26	0.03	0.04
6p25.2-24.3	12–20	0.05	0.00	0.03	0.62	0.07
8q24.13	126–134	0.04	0.03	0.03	0.00	0.37
9p21.3	44–48	0.13	0.23	0.01	0.00	0.00
9q21.31-q22.31	80–98	0.11	0.06	0.41	0.00	0.54
11q23.2-q23.3	120–126	0.26	0.00	0.01	0.00	0.00
12q21.1-q21.33	90–102	0.24	0.03	0.10	0.05	0.27
13q11-q12.3	0–26	0.14	0.51	0.32	0.00	0.01
14q31.1-q32.13	78–96	0.27	0.00	0.10	0.00	0.00
15q13.2-q13.3	20–22	0.22	0.00	0.02	0.00	0.00
15q26.3	122–128	0.01	0.33	0.01	0.00	0.00
19p13.3	10–18	0.02	0.00	0.07	0.69	0.01
19p12-q12	48–54	0.02	0.00	0.03	0.00	0.32
22q13.31	58–60	0.00	0.00	0.03	0.00	0.21

^a^The cM region corresponds to the phenotype with the highest PPL, extending out to cover PPL >10% around that peak for any phenotype. PPLs for additional phenotypes represent the maximum within this region for the given phenotype. ^b^Note that these represent PPLs based on the University of North Carolina (UNC) pedigrees only, and they are therefore not identical to results shown in Figure 2.

Second, ANX, PRS, and SOC show multiple peaks (4 PPLs >20% for ANX, 3 for PRS, 5 for SOC, at a total of 9 loci overall). Apart from 2q37.2-q37.3 (which is best supported by BAP), only 2 loci are strongly supported by 2 or more of these phenotypes (on 9q21.31-22.31, PRS and SOC with slight supporting evidence from ANX; on 13q11-q12.3 ANX and PRS with evidence against linkage for SOC). At the remaining loci, only one phenotype clearly supports linkage; particularly notable in this regard are 9p21.3 and 15q26.3, which are supported by ANX with both PRS and SOC giving evidence against linkage. Thus, in general, there does not seem to be a clear pattern of correlation across these phenotypes. On the contrary, while it is difficult to draw definitive conclusions based on these pedigrees alone, there is some suggestion that the three phenotypes might be picking up different underlying genetic loci. Focusing on the 5 loci with PPL >30% for either ANX, PRS, or SOC (in order to minimize noise due to permutation over the phenotypes): 8q24.13 is supported by SOC with ANX and PRS neutral; 9q21.31-q22.31 is supported by SOC as well as PRS and probably ANX; 13q11-q12.3 is supported by ANX as well as PRS but not SOC; 15q26.3 is supported by ANX but neither PRS or SOC; and 19p12-q12 is supported by SOC but not by ANX, with PRS neutral.

**Figure 5:**
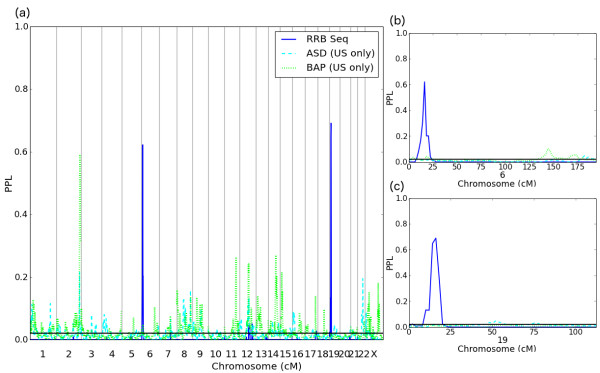
RRB shows two large signals which appear to be independent of ASD or BAP: (a) Genome scan for ASD, BAP and RRB (US pedigrees only); (b) Chromosome 6; (c) Chromosome 19.

Finally, a striking conclusion emerges regarding RRB. RRB itself gives the two highest PPLs seen for any phenotypes (including in the primary analyses based on all of the pedigrees): PPL = 62% at 6p25.2-24.3, and PPL = 69% at 19p13.3. Each of these loci is at best very slightly supported by one additional phenotype, with the remaining phenotypes either neutral or giving evidence against linkage. Particularly notable is that even ASD and BAP do not support these loci (Figure 5). Furthermore, RRB itself gives evidence against linkage at virtually all of the other loci in Table 3 (with the exceptions of 6p25.3-p25.2 and 12q21.1-q21.33 where it is neutral (note that the maximum PPLs at these 2 loci are <30%), and this includes loci supported by BAP. Thus, clear evidence emerges that RRB, while highly informative for linkage in these pedigrees, which were ascertained for the presence of multiple cases of ASD, is in fact segregating independently of both BAP and the remaining subphenotypes, and even ASD itself.

## Discussion

By utilizing a set of extended pedigrees and clinical assessments of a number of phenotypes in both ASD and non-ASD relatives, we have taken an approach that differs from traditional methods used to study the genetics of ASD. This approach has yielded several interesting findings. First, the primary ASD results confirm roles for known ASD loci in extended pedigrees. The four salient loci include the Prader-Willi Angelman region on 15q and the 22q13 deletion syndrome region, both well-established syndromes associated with autistic features in subsets of cases; in addition, 2q37 contains CENTG2, which has obtained modest support as an ASD gene in independent studies [66], and 1p36 is associated with 1p36 deletion syndrome. While considering additional loci highlighted in the analysis of individual pedigrees risks the appearance of 'cherry picking’, here too the findings highlight overlap with previously identified loci, particularly at 8q12.1, which is the ICHD7/CHARGE syndrome region, and Xp22, which covers PTCHD1 [24,67]. While these loci have been highlighted before, they have been discovered primarily through individual case studies or *de novo* CNVs. Our results support the importance of these loci in inherited, highly familial, forms of ASD as well.

Secondly, in aggregate, our results strongly support the presence of substantial between-pedigree locus heterogeneity for both ASD and BAP analyses. Assuming common loci across pedigrees (PPL_POOL_) produced lower PPLs at all loci with PPL >20% compared to allowing for different genetic loci and models across pedigrees (PPL_SEQ_) (PPL_SEQ_ also returned evidence against linkage across a larger proportion of the genome). Moreover, as previously mentioned, support was found for some previously implicated ASD loci in one individual pedigree but not others. This pattern is consistent with results from the CNV analyses published over the last decade that have shown very little sharing of loci across different families. This highlights the need for locus- and gene-discovery methods that are robust to locus heterogeneity, and should inform our interpretation of negative as well as positive findings going forward.

A third notable finding is that, while some peaks are better supported by ASD and others by BAP, in only two cases does a salient peak under one diagnosis show evidence against linkage under the other. In general, adding the BAP to the analysis does appear to confirm the hypothesis that the BAP is of relevance to ASD genetics. Of particular interest, perhaps, are the BAP findings at 2q37.3 and 11q23.2, the first of which contains UBE2F and the second UBE4A. Note that 11q was also reported by Liu et al. [68] in an independent data set.

The fourth interesting finding involves the phenotypes ANX, PRS, and/or SOC, which provided multiple loci of interest. Given correlations across phenotypes within pedigrees, we are sensitive to the speculative nature of any conclusions regarding genetic relationships between phenotypes. However, the patterns of results are consistent with a model in which these sub-phenotypes are segregating independently of one another at some loci, and perhaps independently of BAP in some 'carrier’ relatives, but still be involved in the etiology of ASD if other events or 'hits’ at other loci added to the genetic liability for ASD itself. Such interaction might be specific to particular pedigrees, to particular loci, or may generalize across pedigrees. This seems like a fruitful area of investigation for future family-based genetic studies. The fact that the exploratory phenotypes NWR, RAP, and CLF showed no notable evidence for linkage across the genome also serves as a kind of negative control, suggesting that not all phenotypes conceptually related to the defining features of autism are genetically relevant in these pedigrees. On the other hand, these traits may simply have been less informative in this particular data set; only 2, 5, and 7 families are multiplex for CLF, RAP and NWR, respectively. Additional data collection would be needed to resolve this.

The fifth finding of this study is that RRB appears to be entirely genetically independent of BAP and the remaining secondary phenotypes, yet highly informative in these families, yielding the two largest PPLs seen across all analyses. This is consistent with recent empirical measurement models that suggest that ASD is composed of two key phenotypes that co-occur [69]. Under this model, it remains to be explained why ASD itself does not support linkage to the RRB loci. It is certainly possible that the ASD phenotype alone is simply not informative enough at those loci; for instance, under a two-locus epistasis model, the meiotic information regarding RRB transmission might come primarily from non-ASD individuals, with parents of those who develop ASD tending to be homozygous for RRB genotypes and therefore uninformative. However, this model will remain entirely speculative until it is possible to identify the responsible genomic variants under the peaks.

The strengths of this study include the large number of extended pedigrees with high quality, highly informative marker data, careful attention to measuring multiple phenotypes for both ASD and non-ASD individuals within those pedigrees, and statistical methods, which allowed us to take full advantage of all features of the data. The salient limitation of the study is nevertheless sample size; despite the size of the data set, some phenotypes remained insufficiently informative, and particular relationships among phenotypes remain to be confirmed and further defined. Furthermore, the results themselves suggest considerable heterogeneity between pedigrees, yet each pedigree on its own is not sufficiently large to yield definitive linkage results. Hence, most of the signals reported here remain modest in size and undoubtedly not all of them represent true linkages (although the overlap with known ASD loci supports the efficacy of picking up true ASD genes at several of these moderately supported loci).

Finally, the phenotypes considered here are almost certainly still just proxies for more biologically defined underlying features. This complicates interpretation of results across phenotypes. For example, perhaps the most tantalizing result is the apparent independent segregation of RRB even from ASD within these ASD-multiplex families. This seems contradictory, unless ASD as diagnosed via the Autism Diagnostic Interview and Autism Diagnostic Observation Schedule (ADI and ADOS) is a measure for some aspects of autism but not all. It is worth noting here that the measurement of repetitive behaviors in the RBS-R is not equivalent to what is used for the ASD diagnosis itself; 'repetitive behaviors’ do not constitute a unitary construct, and more finely grained or biologically direct phenotypes that are likely to bring us closer to the complex mechanisms occurring in ASD, would help clarify the ASD-RRB relationship from a genetic point of view [70].

## Conclusions

Overall, our primary conclusions speak to key features of the complexity of the genetic architecture of ASD: i) there appears to be substantial between-family locus heterogeneity; ii) in keeping with previous epidemiologic findings, the BAP does appear to correspond at least roughly to sets of subclinical features segregating with ASD within pedigrees, so that equating non-ASD relatives with non-carriers of ASD genes is not correct in general; and iii) different features of the ASD phenotype appear to segregate independently of one another within these pedigrees, in support of the multiple hit model articulated by Eichler and others [71-73]. If these findings prove correct, they pose a set of challenges to future studies. We believe that these challenges will be met, at least in part, by the study of additional and larger, even more individually informative pedigrees, together with measurement of multiple, and perhaps more biologically direct, phenotypes in both affected and non-affected individuals. Pedigrees of the type needed for these studies are uncommon, but they do exist. Exploiting the many opportunities they provide to further our understanding of the complex genetics of ASD seems both possible and extremely promising.

## Abbreviations

ANX: Anxiety; ASD: Autism spectrum disorder; BAP: Broad autism phenotype; BAP-Q: BAP Questionnaire; CLF: Core language ability; CNV: Copy number variants; MPAS-R: Modified personality assessment schedule revised; MPRS: Modified pragmatic rating scale; MS: Microsatellite; NWR: Non-word repetition; PPL: Posterior probability of linkage; PRS: Pragmatic rating scale; QT: Quantitative trait; RAP: Rapid naming; RRB: Repetitive/ritualistic behavior; SOC: Social functioning.

## Competing interests

The authors declare that they have no competing interests.

## Authors’ contributions

JP, VJV and PS conceived of the study, participated in design and coordination, and performed data interpretation and manuscript preparation; BF, M W-S, IO, MP and AT participated in the design of the study, oversaw data collection, and aided manuscript preparation; YH was involved in development and application of computer programs used for the analyses; KW performed data analysis. All authors read and approved the final version of the manuscript.
